# Association of Faculties of Veterinary Colleges of North America

**Published:** 1895-06

**Authors:** 


Association of Faculties of Veterinary Colleges of North America.—When this Association convenes in September next, as we take it for granted it will, there will be many questions of grave importance come up for consideration. With the changes at the United States College, the Veterinary Department of the Ohio State University and the new laws now in force in Pennsylvania, New York, Maryland and Ohio, with perhaps other States between now and then, all of which will command from it earnest consideration, and we sincerely trust will bring into the organization every other two-year school now in existene.

Apology for Delay.—After waiting many days for the necessary electros for illustration of the history and new home of the New York College of Veterinary Surgeons, we were forced to go to press and delay its publication until the July number, when, in conjunction with that issue, we will publish a brief review of what is going on among the colleges. This will keep our readers posted on every movement of the various veterinary colleges in North America, and better enable a more thorough and just consideration of college affairs at the coming meeting of the United States Veterinary Medical Association.

To Our Subscribers.—With the publication of the June number the Journal will adopt a new rule with its readers. For many years this Journal has continued an unusually lenient course in regard to the payment of subscriptions, in some instances carrying on its books three or four years' back fees, until its losses and uncollected fees have run into several thousand dollars, an amount which has entirely come out of the former editors' pockets. We regret to say that this favoring of its readers has in a large percentage of cases ended in total loss. With the July number we will adopt the plan of rendering a statement at the end of all subscriptions with each current month; a second statement will be rendered during the year, and if said amount is not paid during the yearly term or a satisfactory reason given the managing editor, all journals will be discontinued within thirty days after second notice is sent. We are sure from past experience that it is easier to pay one year than two, and that two years' credit has invariably been followed by a third, and that with it came an indisposition to pay. The Journal has to make settlement of its accounts every thirty days, and we shall spare no expense in making the Journal more readable, attractive, and valuable to its every subscriber. Doing this we feel that we have a just right to expect the help of our subscribers.

